# Brain Plasticity following Intensive Bimanual Therapy in Children with Hemiparesis: Preliminary Evidence

**DOI:** 10.1155/2015/798481

**Published:** 2015-11-10

**Authors:** Maya Weinstein, Vicki Myers, Dido Green, Mitchell Schertz, Shelly I. Shiran, Ronny Geva, Moran Artzi, Andrew M. Gordon, Aviva Fattal-Valevski, Dafna Ben Bashat

**Affiliations:** ^1^Functional Brain Center, The Wohl Institute for Advanced Imaging, Tel Aviv Sourasky Medical Center, 6423906 Tel Aviv, Israel; ^2^Department of Psychology, Gonda Multidisciplinary Brain Research Center, Bar-Ilan University, 5290002 Ramat Gan, Israel; ^3^Sackler Faculty of Medicine, Tel Aviv Sourasky Medical Center, 6997801 Tel Aviv, Israel; ^4^Health and Life Sciences, Oxford Brookes University, Oxford OX3 3FL, UK; ^5^Child Development & Pediatric Neurology Service, Meuhedet, 3350127 Haifa, Israel; ^6^Department of Radiology, Tel Aviv Sourasky Medical Center, 6423906 Tel Aviv, Israel; ^7^Department of Biobehavioral Sciences, Teachers College, Columbia University, New York, NY 10027, USA; ^8^Paediatric Neurology Unit, Tel Aviv Sourasky Medical Center, 6423906 Tel Aviv, Israel; ^9^Sagol School of Neuroscience, Tel Aviv University, 6997801 Tel Aviv, Israel

## Abstract

Neuroplasticity studies examining children with hemiparesis (CH) have focused predominantly on unilateral interventions. CH also have bimanual coordination impairments with bimanual interventions showing benefits. We explored neuroplasticity following hand-arm bimanual intensive therapy (HABIT) of 60 hours in twelve CH (6 females, mean age 11 ± 3.6 y). Serial behavioral evaluations and MR imaging including diffusion tensor (DTI) and functional (fMRI) imaging were performed before, immediately after, and at 6-week follow-up. Manual skills were assessed repeatedly with the Assisting Hand Assessment, Children's Hand Experience Questionnaire, and Jebsen-Taylor Test of Hand Function. Beta values, indicating the level of activation, and lateralization index (LI), indicating the pattern of brain activation, were computed from fMRI. White matter integrity of major fibers was assessed using DTI. 11/12 children showed improvement after intervention in at least one measure, with 8/12 improving on two or more tests. Changes were retained in 6/8 children at follow-up. Beta activation in the affected hemisphere increased at follow-up, and LI increased both after intervention and at follow-up. Correlations between LI and motor function emerged after intervention. Increased white matter integrity was detected in the corpus callosum and corticospinal tract after intervention in about half of the participants. Results provide first evidence for neuroplasticity changes following bimanual intervention in CH.

## 1. Introduction

Cerebral palsy (CP) results from early brain injury, either pre- or perinatal, and affects 2-3 in 1000 children. Approximately 30% of children with CP have hemiparesis, which manifests as motor impairments and weakness on one side of the body and causes substantial functional impairment in day-to-day tasks [1]. Beyond unilateral impairments, children with hemiparesis (CH) also have impairments in bimanual coordination [2].

Several types of intervention have shown success in improving hand function in hemiplegia, the most common of which are constraint induced movement therapy (CIMT), which involves unimanual training [3]. Another less studied therapy is hand-arm bimanual intensive therapy (HABIT) [4, 5], which involves practice of tasks requiring two hands in order to develop use of the affected hand and improve coordination. This type of bilateral intervention has demonstrated substantial benefits in this population [6].

Advanced MRI methods, including functional magnetic resonance imaging (fMRI) and diffusion tensor imaging (DTI), have been shown to provide important information in the evaluation of CP and therapy response assessment [7]. fMRI studies have shown abnormal patterns of activation with a shift towards bilateral activation in CH [8]. Reduced white matter (WM) integrity in corticospinal tracts in the affected hemisphere was also shown using various diffusivity indices, corresponding to severity of hand function impairment [9].

However, little is known about changes in brain structure and function following motor interventions in CH since few studies have used serial imaging. A recent systematic review of the literature found just seven studies, all with small samples (3–10 children) [10]. While research into brain plasticity following rehabilitative interventions in stroke patients is more abundant [11–13], the later time of injury prevents comparison with unilateral CP, which commonly results from pre- or perinatal injury.

The existing studies describing neuroplastic changes following treatment involved either virtual reality intervention [14] or most often unimanual interventions, such as CIMT [10, 15, 16]. A review of the literature reported enlargement of the primary hand motor area (M1) in the affected hemisphere following intervention, with no consistent effects in the less affected hemisphere [10]. A previous study reported change in LI towards unilateral pattern associated with greater improvement in motor function after CIMT treatment [17]. Yet neural changes occurring after bimanual intervention have not been previously described.

The current study sought to characterize brain plasticity following bimanual intervention in children with hemiparesis, via serial behavioral assessment and MR imaging, and to examine the association between brain and behavior changes.

## 2. Subjects and Methods

The study was approved by the Institutional Review Board and National Research Ethics Committee of the hospital, and fully informed consent was obtained from parents and assent from children.

### 2.1. Subjects

12 children with hemiparesis (6 females, mean age 11 ± 3.6 years) were recruited from the Pediatric Neurology Unit at the Tel Aviv Sourasky Medical Center and associated Child Development Centers to a magic-themed HABIT intervention in 2011. The subjects in the current study are part of a larger cohort of CH [6]. Only children with longitudinal imaging were included. Additional inclusion criteria were clinical signs of spastic hemiparesis, attendance in mainstream education, and independence of mobility. Exclusion criteria were any overt seizure activity, initiation of motor therapy or musculoskeletal treatment in the last 6 months, prior surgical intervention, and contraindication to MRI. Level of mobility and functional capacities of the children were confirmed via the Gross Motor Function Classification System (GMFCS) [18] and Manual Ability Classification System (MACS) [19] where higher scores represent greater restrictions to mobility and function. Children were included if GMFCS ≤ II and MACS ≤ III with skills ranging from more mild physical restrictions in mobility and handling of objects to considerable difficulty, thus may use mobility aids and require help to prepare and or modify activities.

### 2.2. Study Set-Up

Overall 12 children underwent the Magic intervention program.

**Figure 1:**
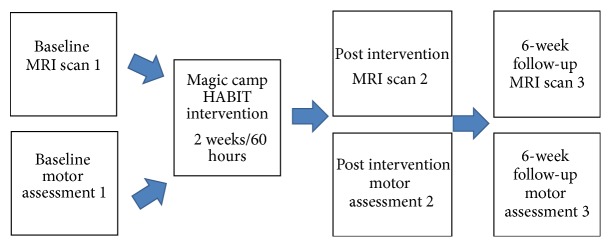
General study set-up for Magic camp HABIT intervention.

Eight children participated in the Magic HABIT day camp intervention (60 hours over 2 weeks) and were assessed for hand motor function on 3 occasions: (1) before intervention, (2) immediately after intervention, and (3) 6 weeks following intervention. Six of them underwent 3 MRI scans at the same time points (see Figure 1). The remaining two children (subjects 2 and 8) underwent only two MRI scans (before and immediately following intervention). Four children participated in an outreach home-based Magic HABIT program (a weekly clinical attendance with 2 hours per day of bimanual training monitored by a weekly diary) for 6 weeks (total of 60 hours) and were assessed for hand motor function on 2 occasions (before and after intervention). These children had only two MRI scans (before and immediately following intervention), except for one child (subject 12) who was excluded after the first scan due to substantial head movements and inability to remain still in the scanner. To set a child friendly atmosphere and improve data quality, training in a mock scanner preceded the MRI scans and during the structural series the children watched an animated movie of their choice. In addition, the child's guardian accompanied the child in all study stages including the scans.

### 2.3. Hand Motor Function Assessment

Children's motor classification included rating according to the MACS and GMFCS. The MACS classifies ability to handle objects in important daily activities across a five-point scale; children at level I handle most objects easily and at level V they are severely limited in their ability [19]. The GMFCS is a measure of spontaneous functional mobility [18].

Hand motor function was assessed at each of the time points with 2 performance tests, assessed by trained therapists and a self-report questionnaire:

1. The Assisting Hand Assessment (AHA; version 4.3): a standardized test of spontaneous use and performance of a weaker/affected hand during bimanual interactions in functional/play based tasks with good reliability and validity [20]. Videos were scored by trained therapists blinded to intervention status.
2. Jebsen-Taylor Test of Hand Function (JTTHF) [21]: a standardized timed test measuring manual dexterity (modified by eliminating the writing task) with reliability and normative data reported for children [22]. A three-minute limit was set for each task with a maximum overall score of 1080 seconds across the 6 tasks.
3. The Children's Hand Experience Questionnaire (CHEQ): a 29-item questionnaire exploring independent participation and skilled use of an affected/hemiplegic hand in daily bimanual activities and reported competence and worry/confidence [23].

Hand motor improvement was defined as follows.

AHA: Least Detectable Difference (LDD) was defined as (1.96*∗*√2*∗*SEM) and was equal to 5 points, representing a clinically meaningful difference for an individual using the Rasch weighted log unit scores. Raw scores are transformed into logits via Rasch analysis to account for different degrees of difficulty of the items. Logits are converted to a 0–100 AHA unit scale with higher scores representing better bimanual skills [20].

JTTHF: percentage of change was determined. Calculation of difference beyond 2 standard deviations of the normative data equated to 20%, representing a clinically meaningful difference.

CHEQ: percentages of change relative to baseline in number of activities performed using 2 hands were calculated and change greater than 20% was considered meaningful [6].

### 2.4. MRI Scanning

Images were acquired on a 3T GE scanner (GE Signa EXCITE, Milwaukee, WI, USA) with training in a mock scanner prior to the first scan.

The MRI protocol included high resolution anatomical 3D fast spoiled gradient echo sequence (FSPGR) (slice thickness/gap = 1/0 mm; field of view (FOV)/matrix: 240 mm/256 × 256; time to repeat (TR)/time to echo (TE) = 8.6/3.3 msec); fMRI performed with *T*_2_^*∗*^-weighted gradient echo echo-planar imaging (GE-EPI) sequence (slice thickness/gap = 3.5/0.3 mm; FOV/matrix = 240 mm/128 × 128; TR/TE/flip angle = 2,250/29 msec/79°); DTI acquired along 19 diffusion gradient directions (*b* = 1000 sec/mm^2^) and one with no applied diffusion gradient (slice thickness/gap = 3/0 mm; FOV/matrix = 220 mm/128 × 128; TR/TE = 11,000/91 msec).

### 2.5. MR Analysis

#### 2.5.1. MRI Motor Paradigm

A block-design fMRI motor task was used in which children clenched and extended all fingers of one hand in synchrony with 2 Hz paced tones. The total task duration was 4 minutes and 48 seconds. There were 18 seconds of silence with one alert beep before the task began to let the children prepare for the motor task, followed by alternations between six epochs of rest, six epochs for right hand, and six epochs for left hand, each lasting 14 seconds. Children were instructed to do their best to move only the affected or less affected hand in isolation. Range of movement was limited to midrange by a soft plastic sponge ball placed in children's palms. Videos were recorded during the fMRI task in order to assess the presence of mirror movements. Mirror movements (MM) were subsequently rated according to the Woods and Teuber scale [24]. On this scale, 0 indicates absence of MM, 1 = barely discernible, 2 = slight but sustained, 3 = strong and sustained, and 4 = movement equal to intended hand.

#### 2.5.2. fMRI Analysis

fMRI analysis was performed with BrainVoyager QX 2 software package (http://www.brainvoyager.com/) and was previously described [9]. Briefly, preprocessing included motion correction (scans with head movement >3 mm were rejected), high-frequency temporal filtering, and removal of low-frequency linear trends. The first six volumes were discarded to allow for stabilization of the signal (to allow for *T*_2_^*∗*^ equilibration effects). Coregistration was performed between anatomical and functional images. Preprocessed functional images were incorporated into the high resolution anatomical images through trilinear interpolation. The coregistered images were not transformed into a standard space but remained in each subject's native space due to the substantial brain abnormalities in this population. fMRI data sets from the 2 or 3 time points (baseline, after intervention, and follow-up) were coregistered to the 3D FSPGR anatomic sequence of each participant from the baseline scan (T1) to allow comparison between activations at the different time points. Three-dimensional statistical parametric maps were calculated separately for each subject using a general linear model (GLM) in which all stimuli conditions were positive predictors. Two contrasts were studied: contrast 1: affected hand versus baseline and contrast 2: less affected hand versus baseline. We used the false discovery rate (FDR) procedures for the selection of thresholds, and the FDR (*q* value) chosen in the present study was 0.05.

Two measurements were extracted.

1. The peak activation in each region of interest (ROI) was detected and a box-shaped volume of 25 voxels was placed around the peak of activation from which beta values were extracted. The beta weights were extracted separately from blocks that included movement of the hand contralateral to the lesion (affected hand) and of the hand ipsilateral to the lesion (less affected hand). The selected ROI beta weights refer to the fMRI activation level and reflect the level in which each of the predictors explain the signal from the specified region. Therefore, the beta weights characterize the level of task-related activity in each region selected.
2. We also applied an additional quantitative measure of lateralization index (LI) using the total number of activated voxels for each region of interest. For each ROI, voxels were collected using all conditions and were compared with the baseline condition with a probability value less than 0.05. LI = (contralateral − ipsilateral)/(contralateral + ipsilateral), where contralateral and ipsilateral equal the total number of voxels activated above threshold in areas around the central sulcus contralateral or ipsilateral to the moving hand. This approach yielded LIs for the motor activation around the central sulcus that ranged from +1 for unilateral activation pattern to −1 for ipsilateral activation pattern (atypical), while values close to 0 reflect more bilateral activation patterns.

#### 2.5.3. DTI Analysis

DTI analysis was performed using DTI Studio software (Johns Hopkins University, Baltimore, MD, USA) as previously described [9]. The diffusion tensor was first estimated on a voxel-by-voxel basis and axial diffusivity (Da), radial diffusivity (Dr), mean diffusivity (MD), and fractional anisotropy (FA) maps calculated. The corpus callosum (CC) and corticospinal tract (CST) were reconstructed using streamline fibre tracking with the Fibre Assignment by Continuous Tracking (FACT) algorithm [9]. Fibre tracking was terminated when it reached a pixel with an FA value lower than 0.25 or when the turning angle was >70°. A single ROI was used to extract the CC via a color coded midsagittal FA image [9, 25, 26]. Further segmentation of the CC into genu, midbody, and splenium was performed based on the Witelson parcellation scheme [27]. A multiple ROI approach was used to extract CST tracts, defining fibres that pass from the unilateral pons through the posterior limb of the internal capsule to the motor and premotor cortex [9]. Mean values of Da, Dr, MD, and FA were calculated for each fibre.

Significant change in diffusivity parameters was defined as follows: changes above 5% were considered significant as previous studies reported changes around 5% following learning interventions, with smaller changes likely to reflect natural variation [28, 29].

### 2.6. Statistical Analysis

Descriptive analysis was performed at a group level to compare the imaging parameters before and after intervention. Improvement on behavioral tests was based on clinical significance as described in Section 2.3 (Hand Motor Function Assessment). For all imaging parameters (beta values, LI, and diffusivity values), mean percent change between pre- and post intervention imaging parameters was computed to assess the change between the different time points and baseline measures. Significant improvement on diffusivity parameters was defined as greater than 5% as described above. Pearson correlations were performed to study the association between the imaging parameters and manual function at the three time points. All statistical analyses were performed using SPSS (Chicago, IL, USA, version 17.0).

## 3. Results

### 3.1. Description of Sample

The study group comprised 12 children (6 males) aged 7–16 years (mean 11 ± 3.6 y), of which nine had right hemiparesis and the remainder had left hemiparesis. MACS scores ranged from 1 to 3, while GMFCS scores ranged from 1 to 2. Two children were born preterm and the rest at term. See Table 1 for subject characteristics.

### 3.2. Behavioral Outcomes

Overall, 11 out of 12 children improved behaviorally after intervention on at least 1 test, and eight children improved on two or more tests (see Table 2). Six out of eight children who were assessed at the third time point maintained improvement at follow-up. It should be noted that the one child who did not improve behaviorally participated in the Magic HABIT camp in the previous year and appeared to experience a ceiling on his hand function progress.

AHA: 7 of 12 children improved significantly (at least 5 points) after intervention. Of these, 3 maintained improvement at follow-up, and 1 further child showed significant improvement only at follow-up.

CHEQ number of independent 2-handed activities: 6 of 12 children improved significantly (at least 20% change) after intervention. Of these, 3 maintained improvement at follow-up, and 1 further child showed significant improvement only at follow-up.

JTTHF reaction time of the affected hand: 8 of 12 children improved significantly after intervention (at least 20% change); 4 children were unable to complete the task within the required time at both time points and thus were scored as not showing improvement. All of the 4 children with the additional assessment at time 3 who improved after intervention maintained the improvement at follow-up.

**Figure 2:**
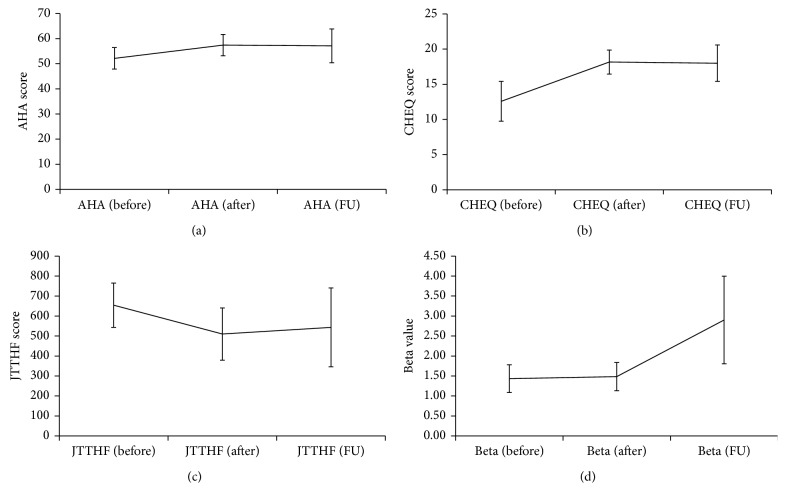
Group means and standard error for (a) AHA, (b) CHEQ number of 2-handed activities, (c) JTTHF of the affected hand, and (d) beta values in affected hemisphere when moving the affected hand. Manual function: before and after, *n* = 12; follow-up, *n* = 8. Betas: before and after, *n* = 7, follow-up, *n* = 4.

At the group level, test scores improved after intervention on all tests, with the improvement tapering off at follow-up, apart from the JTTHF where even greater improvement was seen at follow-up (Figures 2(a)–2(c)). Mean increase in AHA scores from pre- to post intervention was 5.13 ± 4.09 points after intervention and 10.68 ± 13.2 points by follow-up, while mean decrease in response time on the JTTHF was 27.48% ± 36.44% after intervention and 33.6% ± 38.2% from baseline to 6-week follow-up. Mean increase on the CHEQ 2-handed score was 5.58 ± 7.4 points after intervention and 4.57 ± 6.9 points at follow-up.

### 3.3. Motor fMRI Beta Values across MRI Examinations

Overall, level of activation, as measured by beta values, increased after intervention and continued to increase at follow-up (see Figure 2(d)). Mean change in betas in the affected hemisphere when moving the affected hand (contralateral), from pre- to post intervention was 26.14% increase (*n* = 7) and from preintervention to follow-up was 34.75% increase (*n* = 4). In the less affected hemisphere, there was little change in level of activation (ipsilateral to movement of the affected hand) with mean change from pre- to post intervention of −2.4% (*n* = 4). One child (subject 11) was rated as having significant mirror movements (score of 3 on Woods and Teuber scale) which might affect these values. Yet, when excluding this child from the analysis, similar effects were detected with increase of 24% mean percent change from pre- to post intervention (compared to 26.14%) in the affected hemisphere when moving the affected hand and +10% change in the less affected hemisphere when moving the affected hand (compared to −2.4%). There was similarly little change in activation in the less affected hemisphere when moving the less affected hand (contralateral) of −1.5% (*n* = 8) after intervention and 7.6% (*n* = 5) change by follow-up. Only one child had pre- and post intervention activation in the ipsilateral hemisphere when moving the less affected hand.

The most severe case of hemiplegia (case 6), as represented by MACS 3, was the one child who did not respond behaviorally to the intervention. Despite the absence of functional improvement, increases in brain activity as measured by beta levels during hand movement were seen.

### 3.4. Motor fMRI: LI across MRI Examinations

**Figure 3:**
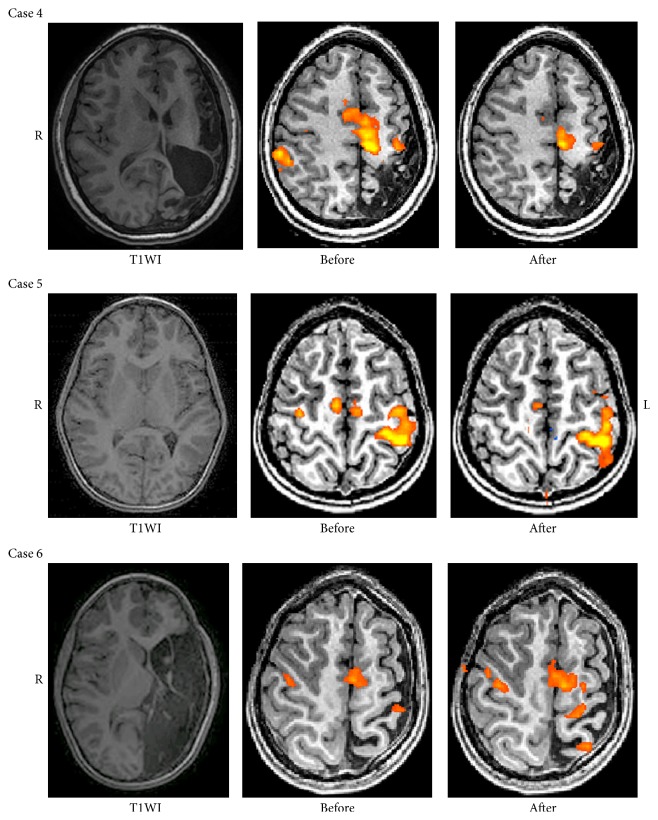
Examples of fMRI motor activation in areas around the central sulcus and supplementary motor area (SMA) for the condition of moving the affected hand. T1WI = T1 weighted imaging. In cases 4 and 5, a more unilateral pattern of activation is seen after intervention. In case 6, there is more activation in the affected hemisphere after intervention.

Motor related activation was seen in the sensorimotor areas around the central sulcus and in the supplementary motor areas (SMA). In general, a shift to a unilateral activation after intervention was detected in some children when moving the affected hand. LI when moving the less affected hand remained stable in all children except one (subject 7), who moved to a more unilateral pattern. Of the 11 children who improved behaviorally, 4 showed improvements in LI on movement of the affected hand, shifting towards a unilateral pattern, or maintained an originally unilateral pattern of activation (subjects 1, 2, 4, and 5) (see Table 3). These improvements were maintained at follow-up (except for 1 child who had no follow-up scan). One child showed improved LI only at follow-up (subject 6). For three children, the LI could not be calculated due to poor data quality of the fMRI scan (subjects 3, 8, and 12). Three children improved behaviorally yet did not have increased LI after intervention, with some demonstrating a pattern of ipsilateral activation (subjects 7, 10, and 11).  Figure 3 shows a graphic presentation of activation in three children, before and after intervention. Subjects 4 and 5 show a change towards unilateral activation after intervention, while only a slight change was evident in subject 6.

### 3.5. Correlations between LI When Moving the Affected Hand with Manual Function at the 3 Time Points

**Figure 4:**
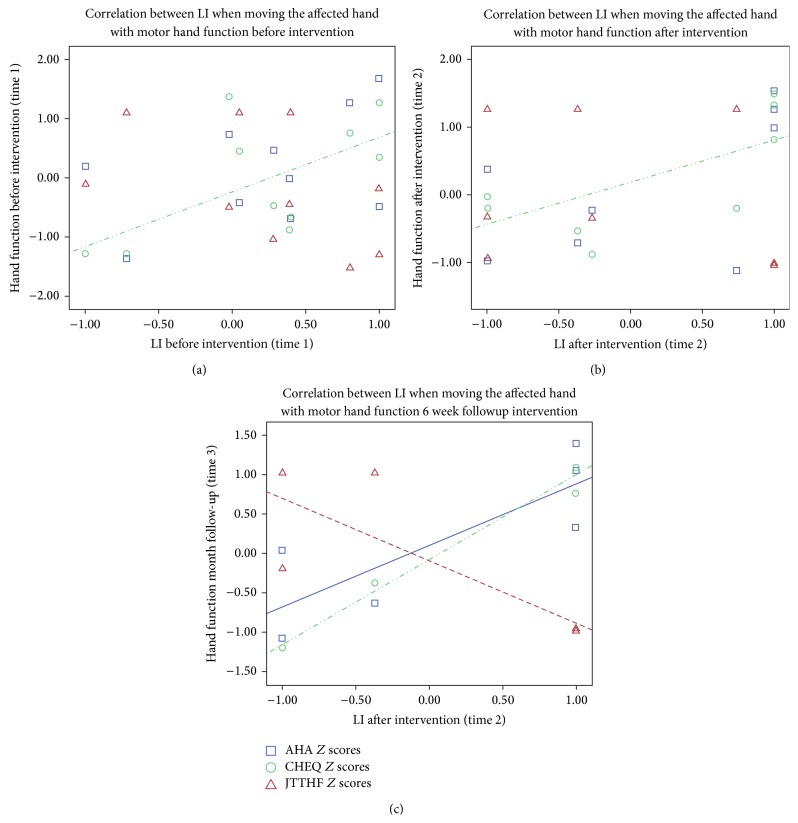
(a) Correlations between LI and manual function at (a) time 1: before intervention, (b) time 2: immediately after intervention, and (c) LI at time 2 and hand function at follow-up. *Y*-axis presents AHA, CHEQ, and JTTHF *Z* scores.

To test the hypothesis that the higher the LI (the more typical/unilateral the pattern of activation), the better the manual function, a correlation analysis between LI and performance was conducted at the 3 time points. Overall, the analysis showed that higher LI values after intervention correlated with better manual skills. A borderline significant correlation (*r* = 0.62, *p* = 0.056) was detected between LI before intervention and preintervention performance on the CHEQ and at post intervention this correlation was significant (*r* = 0.686, *p* = 0.041) (see Figure 4). Similar relationships were not evident between LI and AHA or JTTHF at pre- and immediately post intervention. At follow-up, there was very little variance in LI (4/5 children had values at or close to 1) hampering correlation analysis. Therefore, we examined the correlation between LI after intervention and manual function at follow-up, which enabled us to assess correlation after intervention, either immediately after or at follow-up. In this analysis, strong correlations were detected with all behavioural measures at follow-up: AHA (*r* = 0.820, *p* = 0.046), CHEQ (*r* = 0.941, *p* = 0.005), and JTHHF (*r* = −0.814, *p* = 0.049) (see Figure 4).

### 3.6. DTI Changes in the CC and CST across MRI Examinations

At the group level, no significant changes were detected for the MD and FA values before and after intervention in the CC and in the affected and less affected CST. All changes were under 5% (natural variation). However, on an individual level, several children showed post intervention changes in DTI values associated with improved WM integrity in the CC, affected, and less affected CST greater than those to be expected with natural variation (see Tables 4 and 5). Seven children showed improved diffusivity values in the CC after intervention. One child showed improvements in all segments of the CC. In the CST, 3 children showed improved diffusivity values on the affected side after intervention and 5 children on the less affected side.

### 3.7. Correlations between WM Integrity at the CC and CST with Manual Function at the 3 Time Points

Overall, both before and after intervention, increased WM integrity was related to better hand function. Before intervention, significant correlations were detected between higher WM integrity in the genu and midbody of the CC and better baseline manual function. Lower MD in the genu and midbody was related to higher AHA scores (*r* = −0.58, *p* = 0.05; *r* = −0.75, *p* = 0.008; resp.). Lower MD and higher FA in the genu, midbody and splenium were related to better performance on the JTTHF (genu: FA *r* = −0.618, *p* = 0.032, midbody: MD *r* = 0.668, *p* = 0.025, FA *r* = −0.675, *p* = 0.023; splenium: MD *r* = 0.578, *p* = 0.049; resp.). Higher WM integrity in both the affected and less affected CST was correlated with better unimanual function (JTTHF and FA in the less affected CST: *r* = −0.615, *p* = 0.033; JTTHF and affected CST: FA *r* = −0.667, *p* = 0.035; MD *r* = 0.664, *p* = 0.036).

After intervention, higher WM integrity in the midbody of the CC (reflected by low MD and high FA) was associated with better bimanual function (AHA: MD: *r* = −0.815, *p* = 0.004; FA: *r* = 0.670, *p* = 0.034) and with better unimanual function (JTTHF: MD: *r* = 0.772, *p* = 0.009; FA: *r* = −0.687, *p* = 0.028). No significant correlations were detected between WM integrity and the CHEQ. At follow-up, FA in the genu of the CC was significantly correlated with AHA (FA: *r* = 0.85, *p* = 0.03) and both FA and MD with CHEQ 2 hands (FA: *r* = 0.90, *p* = 0.037; MD: *r* = −0.93, *p* = 0.007). MD and FA in the midbody of the CC were also correlated with AHA (MD: *r* = −0.97, *p* = 0.006; FA: *r* = 0.95, *p* = 0.012) and JTTJF (MD: *r* = 0.93, *p* = 0.021; FA: *r* = −0.88, *p* = 0.048). No significant correlations were detected between WM integrity of the CST and manual function at either post intervention or follow-up.

## 4. Discussion

This study shows the first evidence of brain plasticity in CH following bimanual intervention. Children underwent serial MRI scans including fMRI and DTI and behavioral assessments. Results from this study show changes in levels of activation, in pattern of lateralization, and in WM integrity following intervention. In addition, such changes were correlated with behavioral assessment at all three time points shedding light on possible pathways to explain how behavioral improvement following bimanual intervention is manifested in the brain. Nevertheless, it is important to note that these changes were not detected in all CH regardless of the behavioral gains they showed.

A main finding of this study is a shift towards a more unilateral activation pattern after intervention, reflected by higher LI values. At the group level, abnormal pattern of brain activation was detected at baseline reflected by bilateral activation. In typically developing subjects, motor activation is primarily unilateral, being limited to the hemisphere contralateral to the hand in movement [9]. Following intervention, increased level of activation in the affected hemisphere was detected (manifested by increased beta values) in parallel with the shift in lateralization. We interpreted these findings as indicating neuroplasticity towards a more typical brain activation pattern. These results are in line with a previous study that reported change in LI towards unilateral pattern after CIMT treatment in a small sample (*n* = 4) [17]. A recent systematic review also described several types of brain changes following therapy such as an increase in M1 excitability in subjects with ipsilesional reorganization and a decrease in M1 excitability in subjects with contralesional reorganization [10], indicating treatment-related plasticity. However, brain plasticity is a complex process and changes and varying etiologies, brain injury subtypes, or developmental experiences may have differential effects on neuroplastic changes following intervention. Further studies are needed to address these issues.

The association between LI and manual function became stronger after the intervention with additional associations (with both unimanual and bimanual functions) emerging 6 weeks following intervention. Yet, the improvement in LI at follow-up was not matched by further behavioral improvements at this time. This may reflect the consolidation time of the newly learned skills (plasticity processes) enabling detectable expressions of relations between newly strengthened brain networks and more effective manual function by the end of treatment, and even more so at follow-up. Indeed, children were encouraged to keep practicing their newly learned manual skills; therefore, it may be that the plasticity processes continued during the six weeks enabling greater efficacy in more varied contexts.

The finding that the associations between brain function and structure with behavior were more evident at follow-up may support the dynamical systems theory [31] which postulates that there is a period of instability evident in changing neural networks. From the motor perspective, there are different timescales in the characterization of changing behavior which is reflected in motor learning and development, demonstrated by different learning curves [32]. Nikolai Bernstein's theory of the “degrees of freedom” also relates to a period of enhanced variability in motor learning before the emergence of smooth dynamic motor control [33, 34].

At the group level, no changes in WM integrity of large fibre tracts were seen following intervention, although more than half of the children showed significant change in at least one WM fibre tract in at least one diffusivity parameter. A threshold of 5% was chosen as a significant change based on several studies [28, 29]. Scholz et al. [29] reported on a 6-week juggling intervention in young adults and reported mean increases in FA after training in the order of 5% compared to baseline, with controls showing no significant change. Similarly, a study of young adults learning a new language [28] reported up to 5% change in FA in learners versus controls over a 9-month learning period. Changes in FA in language areas were 1-2% after 1 month, rising to 5% after 9 months; however, controls also showed up to 3-4% change in FA, therefore limiting the reliability of the detected changes. It may be that DTI, despite its sensitivity and important value in learning about WM integrity, is still too crude to measure subtle microstructural changes that may take place following a 2-week intervention. Other diffusion based methods with higher spatial resolution and higher *b* value may be more sensitive to detect such changes [35].

Importantly relationships between WM integrity and manual functions emerged as a function of the intervention, and these relations remained significant and became stronger throughout the study period. Mainly mean diffusivity in the midbody of the CC was associated with better bimanual (AHA) and unimanual (JTTHF) skills. MD values in the midbody of the CC were correlated with the CHEQ at follow-up, and this association was not detected before intervention and was not yet evident directly after intervention. Indeed the CHEQ, which reflects changes in bimanual function in daily activities, was previously reported to significantly improve after intervention [6].

There are several methodological challenges when conducting imaging research in pediatric populations with some challenges specific to CH. There are often problems with data quality caused by excess movements, since children often find it difficult to remain still in the magnet. In addition, many children with CH exhibit additional comorbidities such as attention deficit hyperactivity disorder [36, 37]. Thus, scanning children, especially CH, requires a special set-up. In this study, we used a special set-up which included a practice session in a mock scanner; the presence of the child's guardian during all study stages including the MRI scan; and watching an animation movie of the child's choice during the structural series of the scan. In the current study, we had to exclude only a few data sets due to motion artefacts, but in general the special set-up improved the children's cooperation and data quality.

An additional challenge is that children with movement difficulties frequently show associated head movements with the effort of moving their hands resulting in further motion artefact. Furthermore, the phenomenon of mirror movements which are frequently observed in CH may influence fMRI motor activation measurements. In our study, we recorded videos of the children when performing the motor task and retrospectively could identify mirror movements and take them into account during analysis. Only one child demonstrated significant mirror movements and excluding him did not have a major influence.

Finally, there are methodological challenges in conducting longitudinal MRI studies that quantitatively compare scans acquired at different times. There are many parameters that may affect the signal such as different level of head movement and different level of cooperation and grip force and parameters relating to the magnet. We tried to overcome these problems by using FDR for statistical analysis and by using the laterality index which provides a type of normalization of the fMRI data.

There are several limitations in this study, with some that are inherent to studies of CH. In this study, we had a relatively small sample size since it is difficult to recruit this population and since all children were enrolled to an intervention program which required attendance to a 2-week camp or adherence to a home-based programme. The heterogeneity of the sample due to the varied etiologies underlying CH may affect both the clinical motor features of the children and the type of brain pathology, making it difficult to find a general pattern of plasticity following intervention in this population. Another limitation lies in the hand function assessments that may have impeded our ability to detect change. In particular, we noted a ceiling effect on the JTTHF in which some capacity may have been demonstrated, but unless all items within each task were completed successfully (e.g., all 5 cards turned), a maximum time score of 1080 seconds was awarded and therefore not reflective of more discrete changes.

In conclusion, changes in DTI and fMRI parameters were seen when comparing pre- and post intervention scans in CH following HABIT. Brain plasticity varied in the study group with children showing different patterns of change after intervention. However, change towards a more unilateral brain activation pattern was consistently associated with motor improvements, thereby adding evidence of measurable neuroplasticity changes following bimanual intervention in children with hemiparesis.

## Figures and Tables

**Table 1:** Participant characteristics.

Child	Gender	Age years	Hemiparetic side	GA at birth (weeks)	Birth weight (grams)	Type of injury^*∗*^	Radiological score	GMFCS	MACS
1	M	7.75	L	29	1298	PVL + focal infarct	6	1	1
2	F	7.9	R	31	2100	Infarct	25	2	2
3	M	13	R	40	2700	Infarct at 2.5 years	28	2	3
4	F	16.25	R	Unknown	Unknown	Vascular infarct	19	1	1
5	F	10.6	R	40	3245	Infarct	7	1	1
6	M	9.5	R	39	3470	Intracranial haemorrhage	18	2	3
7	M	9.75	R	31	2000	IVH	15	2	2
8	M	7.8	R	40	3765	Infarct	13	2	3

Total camp	5/8 males	10.3	7/8 R	35.71	2654		16.38	1.63	2.00

9	F	12.9	L	40	3330	Intracranial haemorrhage from TBI at 3 months	14	2	2
10	F	18.6	R	40	2840	Infarct at 7.5 years	21	2	2
11	M	10.8	R	41	2770	Infarct	10	2	2
12	F	7.5	L	40	—	Intracranial haemorrhage after cardiac surgery at 18 months	16	2	2

Total home intervention	1/4 M	12.45	2/4 R	40.25	2980		15.25	2	2

GA: gestational age; PVL: periventricular leukomalacia; TBI: traumatic brain injury; IVH: intraventricular hemorrhage; MACS: Manual Ability Classification System; GMFCS: Gross Motor Function Classification System. Radiological score was calculated according to Shiran et al. [30]. ^*∗*^Injury occurred pre- or perinatally unless specified otherwise.

**Table 2:** Individual behavioral scores for all participants before and after treatment and at six-week follow-up.

Type	Case	AHA1	AHA2	AHA3	CHEQ1	CHEQ2	CHEQ3	JTTHF1	JTTHF2	JTTHF3
		Before	After	Follow-up	Before	After	Follow-up	Before	After	Follow-up

Camp	1	77	80	82^≈^	25	27	28	152.3	38.2^*∗*^	29^≈^
	2	42	41	—	6	17^*∗*^	—	1080	1080	—
	3	27	32^*∗*^	37^≈^	5	15^*∗*^	15^≈^	1080	1080	1080
	4	63	72^*∗*^	63	26	26	22	462.5	48.6^*∗*^	50^≈^
	5	71	76^*∗*^	76^≈^	20	23	25^≈^	68.6	52.8^*∗*^	43^≈^
	6	46	47	46	17	15	12	1080	1080	1080
	7	55	63^*∗*^	58	0	18^*∗*^	12^≈^	612.5	365^*∗*^	442.5^≈^
	8	32	43^*∗*^	38^≈^	0	18^*∗*^	12^≈^	1080	1080	1080

		Pre	Post		Pre	Post		Pre	Post	

Home	9	52	58^*∗*^		4	6^*∗*^		476.1	241.8^*∗*^	
	10	45	54^*∗*^		16	13		578.5	355.6^*∗*^	
	11	59	63		8	17^*∗*^		258	85.8^*∗*^	
	12	57	60		24	23		923.9	612.1^*∗*^	

^*∗*^Significant change between assessments at pre- and post intervention.

^≈^Significant change between preintervention and follow-up assessments.

AHA = Assisting Hand Assessment, CHEQ = Children's Hand Experience Questionnaire, and JTTHF = Jebsen-Taylor Test of Hand Function.

**Table 3:** Lateralization index for all participants across examinations.

Case	Affected LI			Less affected LI			Behavioral improvement
	Before	After	FU	Before	After	FU	
1	1	1	1	1	1	1	UM + BM
2	0.4	0.74	—	1	1	—	BM only
3	—	—	—	—	—	—	BM only
4	−0.02	1	1	1	1	1	UM + BM
5	0.8	1	0.91	1	1	1	UM + BM
6	0.055	−0.37	1	0.97	0.97	0.94	None
7	−0.83	−1	−0.16	0.4	1	1	UM + BM
8	—	—	—	—	—	—	BM only
9	−1	—	—	1	—	—	UM + BM
10	1	−0.27	—	1	1	—	UM + BM
11	0.28	−1	—	0.46	0.41	—	UM + BM
12	—	—	—	—	—	—	UM only

LI: lateralization index; FU: follow-up (6 weeks after intervention); UM: unimanual; BM: bimanual.

**Table 4:** Diffusivity parameters in the corpus callosum: before and after intervention and at six weeks following bimanual intervention.

Case	Genu						Midbody						Splenium					
	MD (×10^−3^ mm^2^/s)			FA (a.u.)			MD (×10^−3^ mm^2^/s)			FA (a.u.)			MD (×10^−3^ mm^2^/s)			FA (a.u.)		
Camp intervention	Before	After	Follow-up	Before	After	Follow-up	Before	After	Follow-up	Before	After	Follow-up	Before	After	Follow-up	Before	After	Follow-up

1	0.85	0.86	0.85	0.63	0.61	0.62	0.85	0.82	0.83	0.59	0.61	0.59	0.8	0.79	0.82	0.67	0.68	0.66
2	1.02	1.05	—	0.56	0.57	—	1.07	1.06	—	0.48	0.48	—	1.12	1.16	—	**0.49**	0.56^*∗*^	—
3	**1.01**	**1.02**	0.95^*∗*^	0.57	0.57	0.57	**1.24**	1.00^*∗*^	1.06^*∗*^	**0.48**	0.52^*∗*^	**0.44**	**0.96**	0.90^*∗*^	0.99	0.63	0.63	0.59
4	0.89	0.88	0.89	0.6	0.6	0.59	**0.93**	0.87^*∗*^	0.88^*∗*^	0.58	0.58	0.56	1.04	1.02	1.19	**0.54**	0.58^*∗*^	0.57^*∗*^
5	0.84	0.86	0.86	0.61	0.61	0.6	0.78	0.79	0.82	0.62	0.61	0.59	0.8	0.8	0.83	0.66	0.65	0.64
6	**0.92**	0.85^*∗*^	0.9	**0.54**	0.59^*∗*^	0.58^*∗*^	0.94	0.93	0.98	0.49	0.5	0.52	**1.11**	1.06^*∗*^	1.01^*∗*^	0.47	0.43	0.45
7	0.97	1.01	1	0.6	0.63	0.57	*∗∗*	*∗∗*	*∗∗*	*∗∗*	*∗∗*	*∗∗*	**0.95**	0.86^*∗*^	0.87^*∗*^	0.67	0.69	0.68
8	0.91	0.92	—	0.59	0.6	—	0.93	0.92	—	0.6	0.6	—	0.84	0.91	—	0.7	0.69	—

Home intervention	Before	After		Before	After		Before	After		Before	After		Before	After		Before	After	

9	0.92	0.99		**0.61**	0.56^*∗*^		0.89	0.85		0.57	0.58		**1.04**	0.95^*∗*^		**0.56**	0.60^*∗*^	
10	0.95	0.92		0.59	0.6		0.84	0.86		0.62	0.59		0.75	0.75		0.7	0.7	
11	0.9	0.96		0.59	0.59		**1.1**	0.93^*∗*^		0.55	0.57		0.82	0.81		0.67	0.69	
12	—	—		—	—													

MD = mean diffusivity (×10^−3^ mm^2^/s); FA = fractional diffusivity (arbitrary units).

^*∗*^Significant improvement >5% from baseline.

^*∗∗*^Due to large lesion size, we were unable to reconstruct this segment.

**Table 5:** Diffusivity parameters in the affected corticospinal tract at 3 time points.

	Affected CST						Less affected CST						
Case	MD (×10^−3^ mm^2^/s)			FA (a.u.)			MD (×10^−3^ mm^2^/s)			FA (a.u.)			Behavior improv.
	T1	T2	T3	T1	T2	T3	T1	T2	T3	T1	T2	T3	
Camp intervention	Before	After	Follow-up	Before	After	Follow-up	Before	After	Follow-up	Before	After	Follow-up	

1	**0.79**	0.75^**∗**^	0.76	0.59	0.6	0.59	0.77	0.74	0.76	0.61	0.62	0.61	Yes
2	*∗∗*	*∗∗*	*∗∗*	*∗∗*	*∗∗*	*∗∗*	**0.8**	0.75^**∗**^	—	**0.56**	0.62^**∗**^	—	Yes
3	0.8	0.84	0.81	0.55	0.55	0.55	**0.8**	0.75^**∗**^	0.8	**0.54**	0.58^**∗**^	0.56	Yes
4	0.79	0.85	0.84	0.58	0.6	0.62^**∗**^	**0.88**	0.75^**∗**^	0.77^**∗**^	**0.62**	**0.58**	0.57^**∗**^	Yes
5	0.73	0.74	0.78	0.61	0.58	0.6	0.72	0.71	0.75	0.6	0.6	0.6	Yes
6	*∗∗*	*∗∗*	*∗∗*	*∗∗*	*∗∗*	*∗∗*	0.76	0.74	0.74	0.59	0.58	0.6	No
7	**0.84**	0.76^**∗**^	0.82	**0.6**	0.66^**∗**^	0.62	0.74	0.73	0.75	0.62	0.6	0.65	Yes
8	0.89	0.87		0.56	0.56		0.73	0.76		0.62	0.62		Yes

Home intervention	Before	After		Before	After		Before	After		Before	After		

9	**0.83**	0.77^**∗**^		**0.6**	0.65^**∗**^		**0.76**	0.71^**∗**^		**0.61**	0.65^**∗**^		Yes
10	0.79	0.82		0.62	0.58		**0.79**	0.71^**∗**^		0.65	0.61		Yes
11	0.81	0.81		0.62	0.6		0.75	0.76		0.63	0.6		Yes
12	—	—		—	—		—	—		—	—		Yes

MD = mean diffusivity (×10^−3^ mm^2^/s); FA = fractional diffusivity (arbitrary units). ^*∗*^Significant improvement = >5% improvement. ^*∗∗*^Due to large lesion size, we were unable to reconstruct this tract.
